# Mesenchymal Stem/Stromal Cells (MSCs) from Mouse Pelvic vs. Long Bones Exhibit Disparate Critical Quality Attributes: Implications for Translational Studies

**DOI:** 10.3390/cells14040274

**Published:** 2025-02-13

**Authors:** Siddaraju V. Boregowda, Cori N. Booker, Jacqueline Strivelli, Donald G. Phinney

**Affiliations:** Department of Molecular Medicine, The Herbert Wertheim UF Scripps Institute for Biomedical Innovation and Technology, Jupiter, FL 33458, USA; siddarajaegowda@gmail.com (S.V.B.); cori.booker96@gmail.com (C.N.B.); strivellij@gmail.com (J.S.)

**Keywords:** mesenchymal stem cells, stromal cells, bone marrow, immune suppression, cell proliferation, critical quality attributes

## Abstract

Mesenchymal stem/stromal cells (MSCs) have been exploited as an experimental cell therapy in a broad array of clinical applications but have underperformed based on results from pre-clinical studies due to gaps in translating pre-clinical findings to human patients. Herein, we isolated mouse MSCs from pelvic bone marrow (BM^P^), a preferred source for human MSCs, and compared their growth, differentiation, and immuno-modulatory activity to those derived from long bone marrow (BM^L^), the traditional source of mouse MSCs. We report that BM^P^-MSCs exhibit significantly enhanced growth kinetics in 5% and 21% oxygen saturation and superior bi-lineage differentiation and hematopoiesis-supporting activity as compared to BM^L^-MSCs. Additionally, we show that TNF upregulates inducible nitric oxide synthase (NOS2) in BM^L^- and BM^P^- MSCs and augments their immune suppressive activity in cell-based assays, while interferon-gamma (INFG) upregulates indoleamine, 2-3, dioxygenase (IDO1) and enhances the immune suppressive activity of only BM^P^-MSCs. These results indicate that mouse MSCs sourced from different bone compartments exhibit measurable differences in critical quality attributes, and these differences are comparable to those observed across species. Based on these differences, BM^P^- MSCs represent a useful resource to model the behavior of human BM-derived MSCs.

## 1. Introduction

Mesenchymal stem/stromal cell (MSC)-based products have been widely tested as experimental therapeutics in humans [1,2,3,4]. However, clinical outcomes have largely been suboptimal compared to those obtained in pre-clinical models [5,6,7,8]. While many factors contribute to this gap in translation, one important confounding variable is the widespread use of mouse MSCs for modeling the potency and mode of action of human MSC-based therapeutics in pre-clinical studies, as these populations exhibit species differences in cellular behaviors. For example, numerous studies have shown that secretion of indoleamine, 2-3, dioxygenase (IDO1), a tryptophan-consuming enzyme highly induced by interferon-gamma (INFG), significantly contributes to the immuno-suppressive activity of human MSCs [9,10,11,12], while inducible nitric oxide synthase (NOS2), which is upregulated by inflammatory cytokines, is necessary and sufficient for mouse MSCs to suppress immune-mediated responses in cell-based assays and in vivo [13,14]. Mouse MSCs have also been shown to express higher levels of interleukin 1 receptor antagonist (IL1RN), a potent anti-inflammatory antagonist of the IL1 family of proinflammatory cytokines, compared to human MSCs [15], and support more robust hematopoiesis in vitro [16,17,18,19,20,21]. However, since primary mouse MSCs exhibit poor growth under standard culture conditions [22,23,24], most laboratories employ populations subjected to long-term culture expansion [25,26,27,28,29,30] or immortalized cell lines as surrogates, which further obfuscates pre-clinical results.

We previously described a method for enriching MSCs using bone marrow from long bones (BM^L^) by immuno-depletion and showed that maintaining cells in a closed, low-oxygen environment maximizes yields while preserving biological activity [31,32,33]. We further showed that oxygen-induced growth inhibition of BM^L^-MSCs is p53-dependent [34] and identified an essential role for basal p53 expression in BM^L^-MSC self-maintenance [35], thereby establishing a mechanistic link between p53, oxygen sensitivity, and cellular immortalization. Since human MSCs are routinely enriched from BM obtained from the pelvic girdle (BM^P^), we questioned whether the bone source confers differences in critical quality attributes. Therefore, we compared the growth kinetics, oxygen sensitivity, bi-lineage potential, and hematopoiesis-supporting activity of mouse MSCs isolated from BM^L^ and BM^P^ via immuno-depletion, which revealed measurable differences in these critical quality attributes. This analysis also revealed differences in sensitivity to cytokines that regulate immune suppressive activity, which correlated with intrinsic differences in immune suppressive activity in a cell-based assay. Together, these data indicate that the skeletal compartment used to source MSCs impacts function to a similar or greater extent than species-related effects, and that BM^P^-MSCs represent a useful resource for modeling the activity of human BM-derived MSCs.

## 2. Materials and Methods

### 2.1. Mice

The methods used to harvest bone marrow and spleens from C57BL/6 mice (The Jackson labs, Bar Harbor, ME, USA, https://www.jax.org/) were reviewed and approved by the Institutional Animal Care and Use Committee at the Herbert Wertheim UF Scripps Institute for Biomedical Innovation and Technology.

### 2.2. Cells

The MSCs were enriched from BM^L^ and BM^P^ obtained from 4–6week-old C57BL/6 mice via immuno-depletion, as described previously [32,35]. Unless indicated otherwise, all cell culture manipulations were performed in a modular airtight chamber (BioSpherix Ltd., Lacona, NY, USA, http://www.biospherix.com/) flushed with 5% CO_2_ and 5% O_2_ balanced with N_2_. To assess growth kinetics, BM^P^- and BM^L^-MSCs were plated at 1000, 2500, or 5000 cells/cm^2^ in complete culture media (CCM; α-minimal essential media containing L-glutamine and supplemented with 10% FBS, 100 U/mL penicillin, and 100 U/m streptomycin) and maintained in 5% O_2_, or switched to 21% O_2_ and then serially passaged at the same initial plating density. Population doubling times were calculated as PD = t log2/(logNt – logNo) where t is time, Nt is the number of cells at time t, and No is the initial number of cells plated. Cumulative cell numbers were determined from the initial plating density and total population doublings for each passage. Cell differentiation was assessed by culturing freshly isolated BM^L^- and BM^P^-MSCs (1000 cells/cm^2^) in 5% O_2_ in CCM for 24 h and then replacing the media with adipogenic induction media (AIM; α-MEM supplemented with 10% rabbit serum, 10^−8^ M dexamethasone, 20 mM 5,8,11,14-Eicosatetraynoic acid, and 25 mg/mL insulin) or osteogenic induction medium (OIM: high glucose DMEM supplemented with 10% FBS, 50 µg/mL L-ascorbic-2-phosphate, 10 mM β-glycerolphosphate, and 10^−8^ M dexamethasone). After 7 days, lipid accumulation was quantified by fixing the cell monolayer in ice-cold methanol for 2 min, staining with AdipoRed^TM^ Assay Reagent (Lonza, Basel, Switzerland) for 10 min, and measuring fluorescence using a SpectraMax fluorescent plate reader (excitation 485 nm; emission 535). At 10 days post-induction, mineralization was quantified by fixing cells for 10 min in 10% neutral buffered formalin, staining cells with 40 mM Alizarin Red-S (pH 4.1) for 5–10 min, removing unbound stains by washing with distilled water, and measuring absorbance at 562 nm. The extent of lipid and mineral staining was normalized to cell numbers by staining cell monolayers with DAPI and quantifying fluorescence using a plate reader.

### 2.3. Colony-Forming Assays

Colony assays were performed using conditioned media (CM) collected from MSC monolayers cultured in serum-free media for 24 h. CM was cleared by centrifugation at 4000× *g* in a centrifugal filter device (Amicon Ultra-4, Burlington, MA, USA) then used to suspend BM^L^ and BM^P^ cells harvested from C57Bl/6 mice. BM^L^ and BM^P^ cells were then mixed with growth factor-supplemented or growth factor-depleted semi-solid media (Methocult, Vancouver, BC, Canada), plated (100,000 cells/dish) in 60 mm dishes, and incubated at 37 °C in a humidified chamber in 5% CO_2_ and 5% O_2_ balanced with N_2_ for 8–10 days. Hematopoietic colonies were scored 10–14 days later, and their identity confirmed by microscopic evaluation.

### 2.4. Immunofluorescent Staining and Flow Cytometry

A single preparation of BM^L^- and BM^P^-MSCs (2.5 × 10^5^ cells/mL) enriched from bone marrow by immuno-depletion and maintained in 5% O_2_ was suspended in Hank’s balanced saline solution (HBSS) and incubated with the following fluorochrome conjugated rat anti-mouse antibodies: FITC-CD11b (Invitrogen, #RM2801, Waltham, MA, USA), FITC-CD34 (BioRad, #MCA1825F, Hercules, CA, USA), FITC-CD45 (BioRad, #MCA1031F), FITC-CD29 (BioLegend, #102206, San Diego, CA, USA), PerCP/Cyanine5.5-CD44 (BioLegend, #103032), PerCP/Cyanine5.5-CD73 (BioLegend, #127214), PerCP/Cyanine5.5-CD105 (BioLegend, #120416) and PE-SCA1 (R & D Systems Inc., #FAB1226P, Minneapolis, MN, USA) for 30 min at 4 °C as described previously [32]. Cells were washed twice with HBSS and analyzed using an EPICS FC 500 FACS scanner equipped with CXP software (Beckman Coulter, Brea, CA, USA). Data were analyzed using FlowJo software 10.9.0 (Tree Star, Ashland, OR, USA).

### 2.5. Quantitative Real-Time PCR (qPCR)

Total RNA was isolated using the RNeasy Kit (Qiagen, Hilden, Germany), converted to cDNA using the Transcriptor First Strand cDNA Synthesis Kit (F. Hoffmann-La Roche, Basel, Switzerland) and amplified by PCR using the TaqMan EZ RT-PCR Kit (Thermo Fisher Scientific, Waltham, MA, USA) according to the manufacturer’s instructions. Reactions were performed on a 7900HT sequence detector (Thermo Fisher Scientific) using FastStart Universal SYBR Green Master Mix (F. Hoffmann-La Roche), and transcript levels were quantified using the relative Ct method with *Gapdh* as an internal control. Primers used for amplification were as follows: *Nos2*, 5′-AGGAGGAGAGAGATCCGATTTAG-3′ and 3′-TCAGACTTCCCTGTCTCAGTAG-5′; *Ido1*, 5′-GCTTCTTCCTCGTCTCTCTATTG-3′ and 3′-CTTTCAGGTCTTGACGCTCTAC-5′; *Il1ra*, 5′-GCTTGAGTCGGCAAAGAAATC-3′ and 3′-GAGAGATGGTCAATGGCAGAA-5′; *LepR* 5′-TGATGTGTCAGAAATTCTATGTGG-3′ and 3′-TGCCAGGTTAAGTGCAGCTAT-5′; *Gapdh* 5′-TCAACAGCAACTCCCACTCTTCCA-3′ and 3′-ACCCTGTTGCTGTAGCCGTATTCA-5′.

### 2.6. Gel Electrophoresis and Western Blot

Protein lysates were prepared using the Qproteome Mammalian Protein Prep Kit (Qiagen) and protein concentrations were determined using the Pierce BCA Protein Assay Kit (Thermo Fisher Scientific). Protein samples (20 µg) were prepared in Laemmli sample buffer (Bio-Rad) containing 5% β-mercaptoethanol, denatured at 95 °C for 10 min, electrophoresed on NuPAGE 10% Bis-Tris gels or 4–12% gradient gels using 1X NuPAGE MES SDS Running Buffer (Invitrogen), and then transferred to 0.45 um nitrocellulose membranes in 1X NuPAGE transfer buffer containing 10% methanol. Membranes were washed with Tris-buffered saline (TBS) for 5 min, incubated in TBS with 0.1% Tween-20 (TBST) and Odyssey^®^ blocking buffer (LI-COR Biosciences, Lincoln, NE, USA) overnight at 4 °C, washed an additional 3x in TBST, and then incubated with antibodies (Santa Cruz Biotechnology, Santa Cruz, CA, USA) against mouse NOS2 (sc-7271; 1:200), IDO (sc-53978; 1:200) or GAPDH (sc-137179; 1:200) in Odyssey^®^ blocking buffer for 2 h at room temperature with gentle agitation. Membranes were washed 5× in TBST and probed with a fluorescent-labeled secondary antibody at a 1:15,000 dilution in Odyssey^®^ blocking buffer for one hour. Blots were scanned using Odyssey^®^ infrared image system (LI-COR Biosciences).

### 2.7. Immune Suppression Activity Assay

Spleens harvested from male C57BL/6 mice (4–6 weeks) were disaggregated, strained through a 10-micron filter, suspended in PBS (3 mL) supplemented with 100 U penicillin and 100 μg of streptomycin, and collected by centrifugation at 900× *g* for 5 min at room temperature. The cell pellet was suspended in 1 mL of ACK lysis buffer, incubated at room temperature for 3 min, and then 6.5 mL of T cell media (RPMI-10 media supplemented with 10% FBS, 1X MEM vitamin solution, 1X MEM NEAA solution, 1X L-Glut, 0.01 M HEPES, 100 U/mL penicillin, 100 U/mL streptomycin, 1 mM Sodium pyruvate), and 10 ng/mL IL2 was added. All media components were purchased from Gibco except FBS (Atlanta Biologics, Norcross, GA, USA) and IL2 (BioLegend; #589102). White blood cells were enriched by centrifugation (800× *g* for 10 min) through Ficoll, collected and washed. Cells (1 × 10^6^) were then suspended in T cell media (100 μL), MSC CM (400 μL) and 10 μL of washed Mouse T-Activator CD3/CD28 beads (Dynabeads^®^, #11452D) at a 1:1 ratio of cells to beads, aliquoted into 24-well plates and cultured at 37 °C in 5% CO_2_ for 3–4 days. Cells were then collected and the beads removed per the manufacturer’s instructions. After washing, cells were suspended in PBS containing 1% BSA and incubated for 10 min with anti-mouse CD16/CD32 Block (1 μL, BD Biosciences, #553142, Franklin Lakes, NJ, USA) and then stained for 15 min at room temperature while protected from light with PE anti-mouse Ki67 (5 μL, BioLegend, #652403) and anti-mouse CD3 (2 μL, BioLegend, #100339) antibodies. Stained cells were then analyzed using an EPICS FC 500 FACS scanner equipped with CXP software (Beckman Coulter) and the percentage of CD3^+^Ki67^+^ cells quantified using FlowJo software 10.9.0 Tree Star). To prepare CM, BM^L^- and BM^P^-MSCs (10,000 cells/cm^2^) were expanded in CCM under low-oxygen (5%) conditions for 48 h. CCM was then replaced with T cell media alone or supplemented with 50 ng/mL IFN, 100 ng/mL TNF, or 50 ng/mL IFN + 100 ng/mL TNF. Then, 48 h later, medium was harvested, centrifuged for 15 min at 500× *g* to remove cell debris and dead cells, and the cleared supernatant was stored frozen. Prior to use, the thawed CM (2 mL) was concentrated to ~400 uL using 10 KDa MWCO Ultra-2 centrifugal filters (Amicon, #UFC201024).

### 2.8. Statistical Analysis

All data were expressed as mean ± standard deviation. Data from a control and a single treated group were compared using Student’s *t* test while data from multiple treated groups were compared to a control using one-way ANOVA and Dunnet’s post hoc test. Differences between groups were considered significant if the *p*-value was <0.05.

## 3. Results

### 3.1. Isolation and Characterization of MSCs

**Figure 1:**
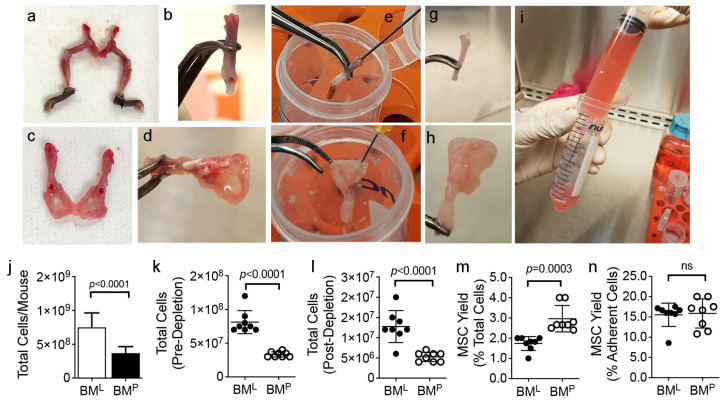
Isolation of MSCs from BM^L^ and BM^P^**.** (**a**,**b**), Representative pictures of dissected hind limbs with attached pelvic girdle after surrounding muscle tissue was removed (**a**) and detached femur with connective tissue removed (**b**); (**c**,**d**), representative pictures of pelvic girdle after surrounding muscle tissue was removed (**c**) and cleaned of attached connective tissue (**d**); (**e**,**f**), purging of bone marrow from the femur (**e**) and pubic (**f**) bones; (**g**,**h**), Femur (**g**) and ilium, ischium, and pubis bones (**h**) purged of bone marrow. Note bones appear opaque; (**i**), single-cell suspension of BM^L^ produced via mechanical dissociation of purged bone marrow plugs using a 28-gauge need. (**j**), total number of BM^L^ and BM^P^ cells recovered on a per-mouse basis; (**k**,**l**), total number of plastic adherent cells (**k**), and MSCs (**l**) recovered pre- and post-depletion, respectively, from BM^L^ and BM^P^; (**m**,**n**), yield of immuno-depleted MSCs as a percentage of total BM cells (**m**) and plastic-adherent cells (**n**). Data are mean ± SD from paired BM isolations (n = 8) and *p*-values are by two-tailed Student’s *t* test. ns = not statistically significant.

To isolate MSCs, the hind limbs (Figure 1a,b) and pelvic girdle (Figure 1c,d) were dissected from surrounding muscle and connective tissue and bone marrow was purged from the femurs, tibiae, fibulae (Figure 1e,g) and the ilium, ischium, and pubis bones (Figure 1f,h). Cells recovered from long (BM^L^) and pelvic bone marrow (BM^P^) were separately pooled (n = 6–10 mice) and disaggregated to create single-cell suspensions (Figure 1i). Total cell yields were significantly greater from BM^L^ vs. BM^P^ on a per-mouse basis (n = 8 preparations) (Figure 1j), which likely reflected anatomical differences in marrow volume. After 5–7d of culture expansion, cell monolayers were harvested and subjected to immuno-depletion [32,33]. The total number of plastic adherent cells (Figure 1k) and MSCs (Figure 1l) recovered pre- and post-depletion, respectively, was significantly greater from BM^L^ vs. BM^P^. Additionally, while MSC yields were greater from BM^P^ vs. BM^L^ (Figure 1m) no significant difference was evident when yields were normalized to the number of plastic adherent cells recovered pre-depletion (Figure 1n). Flow cytometric analysis revealed that MSCs enriched from BM^P^ vs. BM^L^ expressed a similar surface phenotype and were devoid of CD11b-, CD34-, and CD45-expressing cells (Table 1) thereby demonstrating the effectiveness of the immuno-depletion protocol.

**Figure 2:**
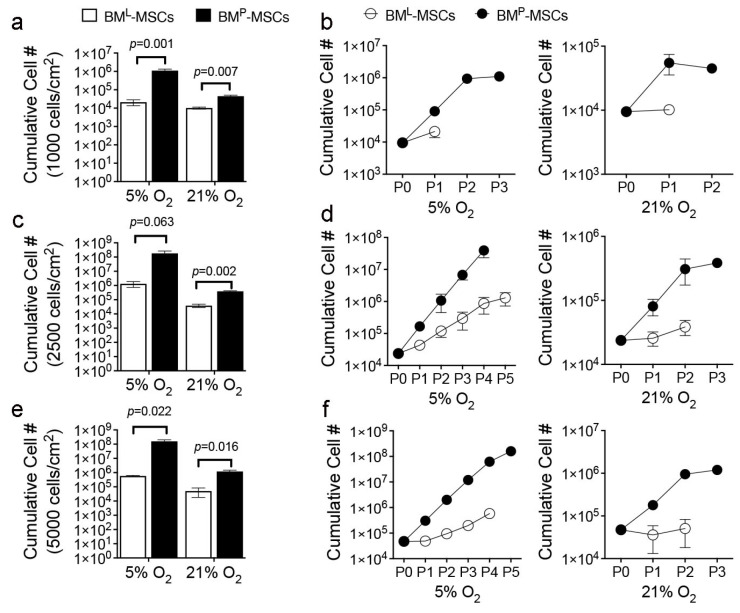
Growth characteristics of BM^L^- and BM^P^-MSCs. (**a**,**c**,**e**), Cumulative yield of BM^L^- and BM^P^-MSCs plated at 1000 cells/cm^2^ (**a**), 2500 cells/cm^2^ (**c**), and 5000 cells/cm^2^ (**e**) and cultured in 5% O_2_ vs. 21% O_2_ for up to four passages (P0-P3); (**b**), cumulative yields of BM^L^- and BM^P^-MSCs from (**a**) as a function of passage number; (**d**), cumulative yields of BM^L^- and BM^P^-MSCs from (**c**) as a function of passage number; (**f**), cumulative yields of BM^L^- and BM^P^-MSCs from (**e**) as a function of passage number. Data are mean ± SD from a single preparation run in triplicate and *p*-values in (**a**,**c**,**e**) by a two-tailed Student’s *t* test. # = number.

Next, we subjected both MSC populations to serial passage in 5% or 21% oxygen saturation using a range of initial plating densities. Under all conditions tested, BM^P^-MSCs exhibited superior growth kinetics compared to BM^L^-MSCs, which reached exhaustion after one or two passages when plated a low density or expanded in 21% O_2_. For example, cumulative yields of BM^P^-MSCs were 52-fold and 4.4-fold greater than BM^L^-MSCs (Figure 2a) when plated at 1000 cells/cm^2^ and expanded under 5% or 21% (Figure 2b) oxygen saturation, respectively. BM^P^- vs. BM^L^-MSC yields were also 140-fold and 10-fold greater at 2500 cells/cm^2^ (Figure 2c,d) and 272-fold and 24-fold greater at 5000 cells/cm^2^ (Figure 2e,f) when expanded in 5% vs. 21% O_2_, respectively (Table 2). Differences in cell yield were attributed to the fact that BM^P^- vs. BM^L^-MSCs consistently attained a high number of population doublings in both 5% and 21% O_2_ at all plating densities evaluated. For example, BM^L^-MSCs underwent 1.05 ± 0.1, 1.64 ± 0.6, and 1.29 ± 0.7 population doublings when plated at 1000, 2500, or 5000 cells/cm^2^, respectively, and cultured in 21% O_2_ while BM^P^-MSCs achieved 2.8 ± 0.7, 4.45 ± 0.8, and 4.97 ± 0.9 doublings. Similarly, BM^L^-MSCs underwent 1.66 ± 0.4, 6.3 ± 1.3, and 4.61 ± 0.8 population doublings when plated at 1000, 2500, or 5000 cells/cm^2,^ respectively, and cultured in 5% O_2_ while BM^P^-MSCs achieved 7.1 ± 1.0, 13 ± 2, and 11.97 ± 0.9 doublings. Although both populations exhibited reduced growth in 21% vs. 5% O_2_, the growth kinetics data indicate that BM^P^- vs. BM^L^-MSCs exhibit lower sensitivity to oxygen-induced stress. 

### 3.2. BM^P^ vs. BM^L^-MSCs Exhibit Superior Differentiation and Hematopoiesis-Supporting Activity

**Figure 3:**
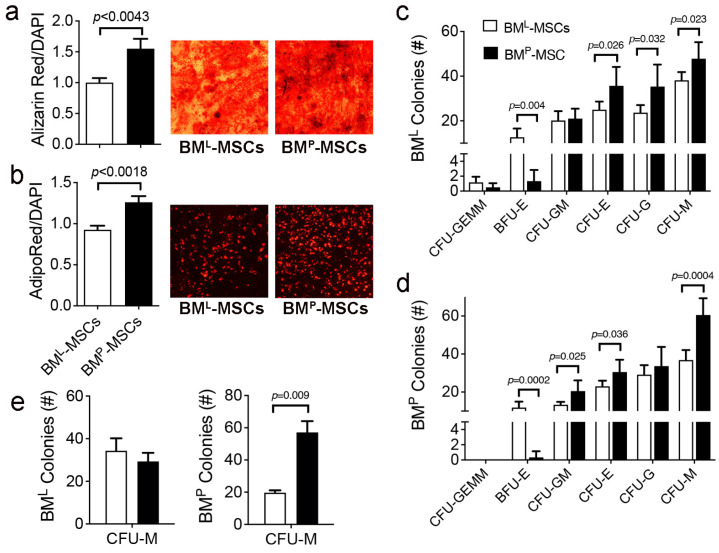
Differentiation and hematopoiesis-supporting capability of BM^P^- and BM^L^-MSCs. (**a**,**b**), Bar graphs showing stimulus-induced osteogenic (**a**) and adipogenic (**b**) differentiation of BM^L^- and BM^P^-MSCs. Representative photos of cell monolayers stained with Alizarin Red S (**a**) or AdipoRed (**b**); (**c**–**e**), total colonies recovered from BM^P^ and BM^L^ cells cultured in growth factor-replete (**c**,**d**) or -depleted (**e**) semi-solid media supplemented with BM^P^- or BM^L^-MSC CM. Data are mean ± SD from two biological replicates run in duplicate and *p*-values by a two-tailed Student’s *t* test. # = number.

Next, using standard cell-based assays, we demonstrated that BM^P^-MSCs yielded significantly more robust osteogenic (Figure 3a) and adipogenic (Figure 3b) differentiation as compared to BM^L^-MSCs. Colony assays also revealed differences in the hematopoiesis-supporting activity of these populations. For example, conditioned media (CM) from BM^P^-MSCs yielded significantly higher numbers of CFU-E, CFU—GM, and CFU-M colonies compared to BM^L^-MSCs when added to BM^L^ cells cultured in growth factor-supplemented methylcellulose (Figure 3c). However, CM from BM^L^-MSCs yielded a significantly higher numbers of BFU-E colonies. Similar results were obtained when studies were repeated using BM^P^ cells cultured in growth factor-supplemented methylcellulose except that BM^P^-MSCs yielded significantly higher numbers of CFU-GM, CFU-E, and CFU-M colonies compared to BM^L^-MSCs (Figure 3d). In the absence of added growth factors, CM from both MSC populations supported expansion of CFU-M colonies only and CM from BM^P^- vs. BM^L^-MSCs yielded more BM^P^-derived CFU-Ms (Figure 3e).

### 3.3. BM^P^- and BM^L^-MSCs Suppress T Cell Proliferation via Different Mechanisms

**Figure 4:**
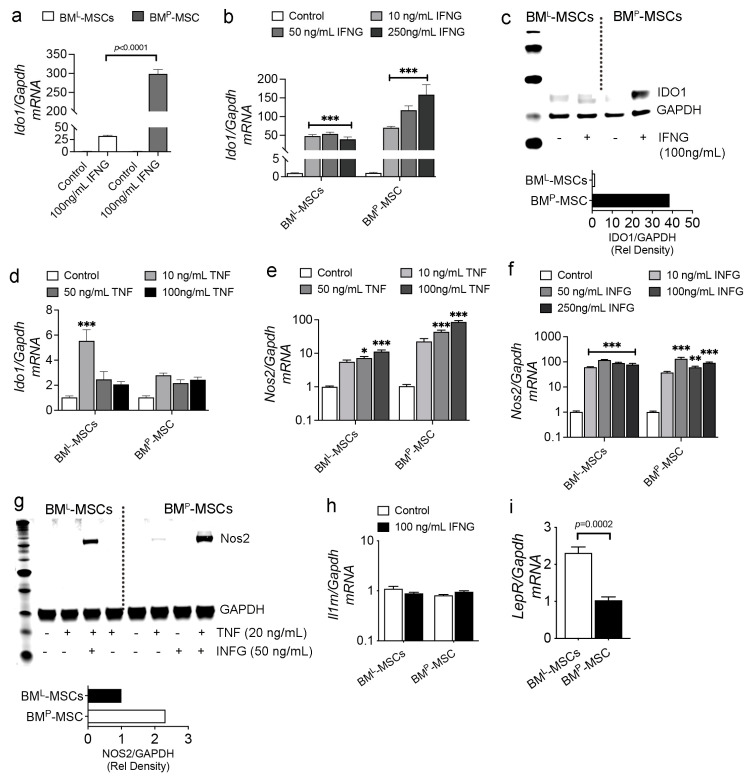
Effect of cytokines on immune effector expression in BM^P^- and BM^L^-MSCs. (**a**), qPCR of *Ido1/Gapdh* mRNA levels in native BM^P^- and BM^L^-MSCs and following treatment with INFG (100 ng/mL) for 24. Data are mean ± SD from biological replicates performed in quadruplicate; (**b**), qPCR of *Ido1/Gapdh* mRNA from MSCs in (**a**) treated with the indicated concentrations of INFG (24 h). Data are mean ± SD from experiments performed in triplicate; (**c**), immunoblot of cell extracts from BM^P^- and BM^L^-MSCs treated without or with INFG (100 ng/mL) for 24 h. Bar graph showing IDO1/GAPDH band intensity for INFG-treated samples quantified via Adobe Photoshop; (**d**), qPCR of *Ido1/Gapdh* mRNA levels in cells from (**a**) treated with the indicated concentrations of TNF for 24 h. Data are mean ± SD from experiments performed in quadruplicate; (**e**,**f**), qPCR of *Nos2/Gapdh* mRNA levels in cells from (**a**) treated without or with the indicated concentrations of TNF (**e**) or INFG (**f**) for 24 h. Data are mean ± SD of experiments performed in triplicate; (**g**), immunoblot of cell extracts from BM^P^- and BM^L^-MSC treated without or with the indicated concentrations of TNF and INFG for 24 h. Bar graph showing NOS2/GAPDH band intensity for TNF + INFG-treated samples quantified via Adobe Photoshop; (**h**,**i**), qPCR of *Il1ra/Gapdh* (**h**) and *LepR/Gapdh* (**i**) mRNA levels in native BM^P^- and BM^L^-MSCs (**h**,**i**) or after 24 h treatment with 100 ng/mL INFG. For a and i, *p*-values are by Student’s *t* test and for b and d–f by one-way ANOVA with Dunnet’s post hoc test for multiple comparisons with * *p* < 0.05, ** *p* < 0.01, and *** *p* < 0.001 vs. controls.

To assess sensitivity to biologics known to regulate immune suppressive activity, we initially cultured both MSC populations in media supplemented with INFG (100 ng/mL) for 24 h and then quantified *Ido1* mRNA levels by qPCR. Herein, IDO1 levels were induced by 30- and 293-fold in BM^L^- and BM^P^-MSCs, respectively (Figure 4a). To confirm this result, we cultured cells in varying concentrations of INFG, which revealed clear differences in responsiveness. For example, *Ido1* mRNA levels were upregulated to a significantly greater extent in BM^P^- vs. BM^L^-MSCs (53 vs. 158-fold) and INFG action was dose-dependent only in BM^P^-MSCs (Figure 4b). Consistent with this result, immunoblot analysis confirmed that INFG induced IDO1 protein expression by 39-fold in BM^P^-MSCs and 1.4-fold in BM^L^-MSCs (Figure 4c). When MSCs were cultured in media supplemented with varying concentrations of TNF (10–100 ng/mL), *Ido1* levels were upregulated to a significant extent compared to untreated controls, but the magnitude of induction was significantly weaker (2 to 5-fold) compared to that obtained with INFG (Figure 4d). Gene expression studies further revealed that *Nos2* mRNAs were significantly upregulated in both MSC populations by TNF (Figure 4e) and INFG (Figure 4f). However, immunoblot analysis revealed that INFG failed to induce detectable levels of NOS2 protein in both MSC populations, while TNF induced low levels of NOS2 protein in BM^P^-MSCs (Figure 4g). Treatment with TNF and INFG in combination yielded robust NOS2 induction in both MSC populations, although levels were ~2-fold higher in BM^P^- vs. BM^L^-MSCs (Figure 4g). We further showed that both MSC populations expressed equivalent levels of *Il1rn* mRNA and that its expression was unaltered following treatment with INFG (Figure 4h). BM^L^-MSCs also expressed significant higher levels of the skeletal stem cell marker *LepR* [36] compared to BM^P^-MSCs (Figure 4i).

**Figure 5:**
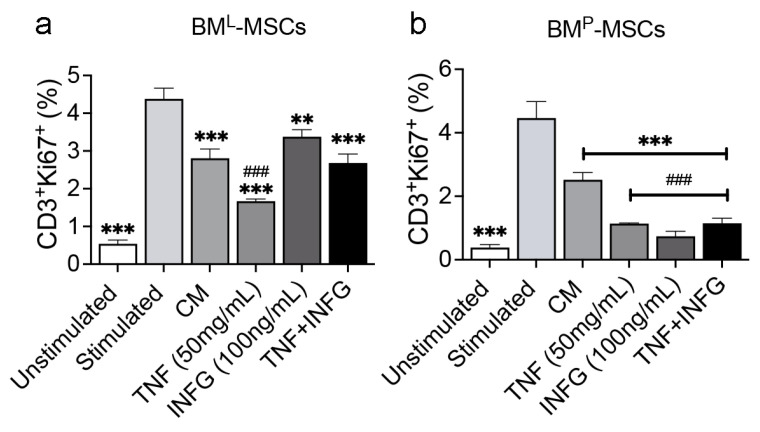
Immuno-suppressive activity of BM^P^- and BM^L^-MSCs. (**a**,**b**), Bar graphs showing percentage of CD3^+^Ki67^+^ splenocytes in unstimulated cultures, after stimulation with anti-CD3/CD28 activator beads, and in the presence of CM from BM^L^-MSCs (**a**) and BM^P^-MSCs (**b**) alone or pre-treated for 48 h with TNF(50 ng/mL), INFG (100 ng/mL), or TNF + INFG. Data are mean ± SD of experiments performed in duplicate and *p*-values by two-way ANOVA with Dunnet post hoc test. ** *p* < 0.01, *** *p* < 0.005 vs. stimulated; ^###^ *p* < 0.005 vs. CM.

To determine whether differences in cytokine sensitivity impact immune suppressive activity, we interrogated the ability of CM from BM^L^- and BM^P^-MSCs to suppress proliferation of splenic lymphocytes following T cell receptor activation by quantifying changes in the percentage of CD3^+^Ki67^+^ cells by flow cytometric analysis. Herein, stimulation of splenocytes with T-Activator CD3/CD28 beads significantly increased the percentage of CD3^+^Ki67^+^ cells compared to unstimulated populations, and co-culture of stimulated splenocytes with CM from native BM^P^- and BM^L^-MSCs resulted in a significant reduction in CD3^+^Ki67^+^ cell abundance, confirming that both MSC populations have intrinsic immune suppressive activity (Figure 5). CM harvested from BM^L^-MSCs pre-treated with TNF (50 ng/mL) suppressed T cell proliferation to a significantly greater extent than CM from native BM^L^-MSCs. However, the activity of CM from INFG (100 ng/mL) or INFG + TNF treated BM^L^-MSCs did not differ significantly from BM^L^-MSC CM alone (Figure 5a). Conversely, CM from BM^P^-MSCs pre-treated with TNF, INFG, and TNF + INFG suppressed T cell proliferation to a significantly greater extent than CM from native BM^P^-MSCs (Figure 5b). Together, these data demonstrate that the ability of TNF and INFG to differentially regulate expression of NOS2 and IDO1 in BM^L^- and BM^P^-MSCs also extends to immune suppressive activity in cell-based assays, and the activity of INFG in BM^P^-MSCs reflects that reported for human BM-derived MSCs.

## 4. Discussion

In this study, we successfully adapted a well-established immuno-depletion protocol to enrich primary MSCs from BM^P^ in high yields. Subsequent comparison of MSCs derived from BM^P^ and BM^L^ revealed important differences in several critical quality attributes. For example, BM^P^-MSCs consistently exhibited superior growth kinetics compared to BM^L^-MSCs at all plating densities evaluated when cultured in 5% or 21% oxygen saturation. Based on our previous findings demonstrating that BM^L^-MSCs are highly sensitive to oxygen-induced growth arrest via a p53-dpedent mechanism [34,37], these data indicate that BM^P^-MSCs are inherently less sensitive to oxygen stress than BM^L^-MSCs. Future studies aimed at quantifying impacts of oxygen exposure on cell survival, mitochondrial function, and p53 expression are needed to provide mechanistic insight into how populations manage such stress. Importantly, while human BM-derived MSCs exhibit robust growth in room air (21% O_2_), these conditions induce measurable oxygen-induced stress, resulting in impaired mitochondrial function [38]. Therefore, based on growth kinetics, BM^P^-MSCs behave more similarly to human BM-derived MSCs than compared to BM^L^-MSCs. We also showed that BM^P^- vs. BM^L^-MSCs exhibit more robust stimulus-induced adipogenic and osteogenic differentiation, consistent with previous studies indicating that oxygen stress, which negatively impacts cell fitness, influences the magnitude and trajectory of mouse MSC cellular differentiation [34].

Our study also revealed significant differences in the sensitivity of BM^P^- and BM^L^-MSCs to cytokines that regulate immune suppressive activity. Specifically, we showed that TNF and INFG in combination significantly upregulated NOS2 mRNA and protein expression in BM^L^- and BM^P^-MSCs and enhanced their ability to suppress the proliferation of splenic T cells in vitro. These data are consistent with earlier studies examining NOS2 transcriptional regulation and mode of action in mouse MSCs [14,39,40,41]. Alternatively, while IDO1 is known to confer immune suppressive activity onto human BM-derived MSCs [42], several studies have shown that it is dispensable for this activity in mouse MSCs. For example, Lanz et al. [13] showed that while IDO1 is induced by proinflammatory stimuli in cells derived from BM^L^, it did not promote tryptophan catabolism or mediate suppression of activated myelin-specific T cells in vitro. These authors further showed that wild-type and IDO1^−/−^ mouse MSCs were equally effective in ameliorating disease severity in a mouse model of experimental autoimmune encephalomyelitis. Similarly, Ren et al. [14] demonstrated that the ability of mouse MSCs to suppress proliferation of anti-CD3-activated splenocytes in culture was not impacted by indomethacin, a PGE2 inhibitor, or L-methyl-DL-typtophan, an IDO1 inhibitor. Therefore, our data, in demonstrating that INFG does not influence IDO1 expression or the immune suppressive activity of BM^L^-MSCs, replicate previous findings.

Alternatively, we show that BM^P^-MSCs upregulate both IDO1 and NOS2 when challenged with the appropriate licensing cocktails, and that INFG does augment the immune suppressive activity of cells in vitro. Since previous studies have not described the isolation and characterization of mouse MSCs from BM^P^, these findings do not contradict prevailing knowledge but add new insights into how the marrow source influences MSC function. For example, based on the inducibility of IDO1 and NOS2 by inflammatory cytokines in MSCs, Su et al. [43] classified MSCs into two distinct groups; IDO1 utilizers (monkey, pig, human) and NOS2 utilizers (mouse, rat, rabbit, hamster). Our findings challenge this classification, as our data suggest that BM^P^-MSCs also utilize IDO1. Other studies have challenged this dogma by showing that NOS2 is dispensable for the immune suppressive activity of mouse MSCs. For example, Bouffi et al. [44] reported that the ability of mouse MSCs to suppress proliferation of concavalin A-stimulated mouse splenocytes in vitro was only partially dependent on NOS2 and prominently regulated via IL6-mediated induction of PGE2. Similarly, Philipp et al. [40] reported that IL6 secreted by mouse MSCs is the main driver of macrophage polarization under inflammatory conditions. A caveat to these and other studies involving mouse MSCs it the fact that no standard methods exist to isolate cells despite evidence that culture expansion greatly influences their intrinsic qualities. For example, most studies enrich MSCs from BM^L^ by plastic adherence and obtain a homogenous population free of hematopoietic markers by subjecting bulk populations [40,44] or individual clones [14,41] to long-term expansion, which invariably yields MSCs that are insensitive to oxygen stress, as evidenced by their robust and prolonged proliferation in 21% O_2._ These approaches differ from our method of isolation, which employs immuno-depletion as opposed to long-term culture expansion and maintains cells in a closed, low-oxygen environment to prevent exposure to growth-restrictive conditions that drive cellular immortalization [34,45].

## 5. Conclusions

In summary, our data reveal clear differences in critical quality attributes of mouse MSCs derived from different marrow compartments, and these differences are comparable to that observed across species. For example, our data indicate that the growth kinetics, oxygen sensitivity, and immuno-modulatory activity of BM^P^-MSCs more closely model those of human BM-derived MSCs than their BM^L^-MSC counterparts. Accordingly, the marrow source should be considered as an important variable in designing experiments employing and interpreting results obtained with mouse MSCs.

## Figures and Tables

**Table 1:** Surface phenotype of BM^L^- and BM^P^-MSCs based on flow cytometric analysis.

Antigen	BM^L^-MSCs (% Positive)	BM^P^-MSCs (% Positive)
CD11b	4.17	1.17
CD34	1.19	2.12
CD45	1.99	1.61
SCA1	96.7	97.2
CD29	99.7	99.8
CD44	99.7	99.7
CD73	99.9	99.5
CD105	46.9	46.7

**Table 2:** Impact of oxygen saturation on expansion of BM^P^- and BM^L^-MSCs.

O_2_ Saturation	Plating Density (Cells/cm^2^)	BM^L^-MSCs (Total #)	BM^P^-MSCs (Total #)	Fold Difference (BM^P^/BM^L^)	*p*-Value
5%	1000	2.12 ± 0.75 × 10^4^	1.10 ± 0.22 × 10^6^	52	0.014
	2500	1.29 ± 0.58 × 10^6^	1.82 ± 0.83 × 10^8^	140	0.063
	5000	5.83 ± 0.54 × 10^5^	1.58 ± 0.42 × 10^8^	272	0.022
21%	1000	1.01 ± 0.12 × 10^4^	4.48 ± 0.05 × 10^4^	4.4	0.007
	2500	3.85 ± 010 × 10^4^	3.86 ± 0.37 × 10^5^	10	0.002
	5000	5.07 ± 3.2 × 10^4^	1.19 ± 0.27 × 10^6^	24	0.016

Paired preparations of BM^P^- and BM^L^-MSCs were plated at the indicated densities in 5% or 21% oxygen saturation, expanded up to four passages (P0-P3), and the cumulative cell number (#) was determined by counting. Data are mean ± SD from three technical replicates and *p*-values determined by Student’s *t* test.

## Data Availability

The data presented in this study are available on request from the corresponding author.
